# Nurses’ representations about partner participation in prenatal care: perspective on masculinities

**DOI:** 10.1590/0034-7167-2024-0160

**Published:** 2025-03-14

**Authors:** Lise Maria Carvalho Mendes, Nayara Gonçalves Barbosa, Edson Silva do Nascimento, Ana Karina Bezerra Pinheiro, Flávia Azevedo Gomes-Sponholz

**Affiliations:** I Universidade Federal do Amapá. Macapá, Amapá, Brazil; II Universidade de São Paulo. Ribeirão Preto, São Paulo, Brazil; III Universidade de São Paulo. São Paulo, São Paulo, Brazil; IV Universidade Federal do Ceará. Fortaleza, Ceará, Brazil

**Keywords:** Prenatal Care, Paternity, Men’s Health, Prenatal Education, Gender Perspective, Atención Prenatal, Paternidad, Salud del Hombre, Educación Prenatal, Perspectiva de Género

## Abstract

**Objectives:**

to understand nurses’ representations about partner participation in prenatal care.

**Methods:**

an exploratory, descriptive, qualitative study, carried out in four Basic Health Units, located in two municipalities in the Brazilian Amazon region. Open-ended interviews were conducted with 11 nurses who had been working in prenatal care for at least six months, between January and July 2023. Professionals who were on sick leave or vacation were excluded. Data organization was carried out through content analysis, and theoretical analysis was based on the gender category.

**Results:**

perceptions of partner participation were observed based on representations of hegemonic masculinity, but indicators of a paradigm shift regarding paternity were also observed.

**Final Considerations:**

professionals reinforce traditional gender roles, treating care as something inherent to women. It was also observed that the category overload leads to the perpetuation of biomedical models of care.

## RESUMEN

**Objetivos:**

comprender las representaciones de enfermeras sobre la participación de los compañeros en el cuidado prenatal.

**Métodos:**

estudio exploratorio, descriptivo, cualitativo, realizado en cuatro Unidades Básicas de Salud, ubicadas en dos municipios de la región amazónica brasileña. Se realizaron entrevistas abiertas a 11 enfermeros que trabajan en el prenatal durante al menos seis meses, entre enero y julio de 2023. Se excluyeron los profesionales que se encontraban de baja por enfermedad o vacaciones. La organización de los datos ocurrió a través del análisis de contenido y el análisis teórico se basó en la categoría de género.

**Resultados:**

se observaron percepciones de participación de la pareja basadas en representaciones de masculinidad hegemónica, pero también se observaron indicadores de cambios de paradigma respecto de la paternidad.

**Consideraciones Finales:**

los profesionales refuerzan los roles tradicionales de género, tratando el cuidado como algo inherente a las mujeres. También se observó que la sobrecarga de la categoría conduce a la perpetuación de modelos biomédicos de atención.

## INTRODUCTION

Paternal relationships in the contemporary family have undergone significant transformations, especially since the 1970s, with the advent of access to contraceptive methods and the mass insertion of women in the work environment outside the home, in addition to the dissemination of feminist studies on gender roles^(1)^. These transformations have driven the emergence of a new expression of the male role in society^(2)^. The expression and need for these new models of expressing the male role also emerge from evidence of negative results regarding the hegemonic masculinity model that was in force previously and that reverberates in the representations of the roles taken over by men in self-care and in the current paternity models^(3)^.

Traditional masculinity models and the way male socialization occurs weaken or even distance men from concerns about self-care and seeking healthcare services^(2-4)^. Denial of fear, exposure to risk and silence about physical and psychological pain are considered traits of hegemonic masculinity, valued for being diametrically opposed to the fragility and emotionality characteristic of hegemonic femininity. Such elements justify the low demand by men for Primary Health Care (PHC) services, the high rates of morbidity and mortality due to preventable diseases or involvement in situations of violence^(2,3)^. The perspective of care can follow a positive path when it incorporates the idea that men are also allowed to pay attention to themselves^(4)^.

Changes in gender roles in society can generate short, medium and long-term benefits, which is why they are important for scientific debate. Among these benefits, it is observed that women who receive routine prenatal care from their partners have fewer complications during labor, childbirth and postpartum, and improved emotional aspects during pregnancy^(5)^. A meta-analysis found that encouraging active paternity, through sharing responsibilities from pre-conception appointments to childcare, has an impact on the bond formed with children, positively impacting biopsychosocial aspects and improving outcomes for the entire family in lowand middle-income countries^(6)^. Thus, it was found that there is a need for a paradigm shift in order to achieve better health indicators, strengthened family ties and a less conflict-ridden society^(3)^.

Brazil was a pioneer in Latin America in creating a public policy aimed at men’s health, one of whose objectives was to expand access and support for men in healthcare services and programs by including the topic of paternity and care^(7)^. Actions aimed at reproductive planning as an essential strategy to improve care during pregnancy, labor and childbirth, with the inclusion of partners, gained prominence in 2016, with the formulation of Prenatal Partner Strategy. This strategy constitutes a gateway for men to the healthcare service network, taking advantage of their presence in prenatal appointments to offer routine exams and educational activities, seeking to provide comprehensive care for this population^(8)^.

It is worth noting that greater attention to this issue could contribute to achieving the 2030 agenda Sustainable Development Goals (SDGs), especially the third, related to good health and well-being, with an emphasis on targets of reducing premature deaths from chronic non-communicable diseases (NCDs), maternal mortality, infant mortality, substance abuse, ensuring universal access to sexual and reproductive healthcare services, as well as the fifth, which aims to reduce gender inequalities^(9)^. Man participation in prenatal appointments provides an opportune moment that can help in the process of constructing new representations regarding paternity and new models of masculinity, bringing men closer to healthcare services and care practices^(8)^.

However, the literature points to some factors that prevent men from attending prenatal care appointments, such as issues related to gender roles, opening hours of Basic Health Units (BHU), lack of educational actions aimed at partners, and lack of encouragement by health teams for partners to participate during appointments and exams^(10)^. Developing strategies and tools to identify the presence of partners in prenatal appointments and in the care environment has been a global challenge^(6,11)^.

It is important to emphasize that the nursing team has a categorical role in the fight for integrating men during the pregnancy-puerperal cycle as part of nursing care and, consequently, as a way of promoting comprehensive care, through welcoming, qualified listening and encouraging the connection of partners with healthy parenting. Due to the relevance of this topic, it is imperative to address nurses’ representations on partner inclusion and participation in prenatal appointments, since nursing, as a science, is a formulator of strategies that enable integration and promote, above all, improvement of care.

## OBJECTIVES

To understand nurses’ representations about partner participation in prenatal care.

## METHODS

### Ethical aspects

This study complied with national and international regulatory requirements for research involving human beings, in accordance with Resolution 510/2016 of the Brazilian National Health Council (CNS - *Conselho Nacional de Saúde*). It obtained a favorable opinion in December 2022 from the Research Ethics Committee of a public university in the far north of Brazil. Participants consented to participate in the study by signing the Informed Consent Form (ICF). The interviews were coded using the letter N (nurse), preceded by an ordinal number. In compliance with methodological rigor, the COnsolidated criteria for REporting Qualitative research (COREQ) guidelines were used.

### Study design and theoretical-methodological framework

This is an exploratory, descriptive study with a qualitative approach, of the strategic social research type, in which the gender category was used as an analytical lens. This category, in the foreground, focuses on understanding that there is a distinction between what is biological sex and what is social sex. The first refers to anatomical-physiological, therefore biological, differences that exist between men and women, and the second concerns the expression that these differences in biological sex assume in different societies^(12)^. Thus, gender can be understood as the constitutive element of social relations based on differences perceived between sexes^(13)^.

In this regard, it is worth noting that there are several ways of performing the roles attributed to men, i.e., masculinity, which vary according to cultural aspects and intersections of class, race and generation^(4)^, structural specificities between men and women due to differences in demands that fall on each role. Among the different ways of expressing masculinity, the predominant one is that which arises from a patriarchal culture that judges men as superior to women^(14)^. The validation of this type of masculinity is limited to characteristics that must be present in a man, such as being assertive, not showing feelings, engaging in risky behavior, having sexual relationships with as many women as possible, not engaging in activities of caring for oneself, one’s children and the home, among other aspects that are harmful to biopsychosocial health^(4,14)^.

As with masculinities, there are different ways of exercising paternity, and its approach is not a universally determined issue^(4)^. It is recognized that this historically and socially constructed determination influences the perception, visibility, practices and coping with dominant models of masculinity and paternity. The unconscious that governs relations between sexes, both in its individual origin and in its collective evolution, is deeply linked to a set of fields and agents that compete for the monopoly of the legitimate definition of sexual practices and discourses, imposing these definitions^(15)^. This representational unconscious is dispersed in society and may also be present in discourses and/or practices of State care agents. Therefore, it is considered that the gender social category can determine the historical and social construction of the representations present in discourses on partner participation in prenatal care by nurses.

### Study setting and data source

The research was conducted in four BHU located in two municipalities in the far north of the Brazilian Amazon. The methodological perspectives established by Bourdieu were used, and the municipalities where the data were collected were not identified so that it would not be possible to identify participants and, in this way, protect and ensure the confidentiality collected in a relationship of trust, and mitigate the constraints to which their words could be exposed^(16)^. Study participants were 11 nurses who had been working in prenatal care for at least six months. Professionals who were on sick leave or vacation were excluded. The sample was intentional. Thus, the BHU were chosen because they are strategic units in the municipalities where the data were collected, and all 14 nurses from these BHU were invited to participate in the study. One nurse was on maternity leave at the time, and two others refused to participate in the study due to task overload and incompatible schedules.

### Data collection, organization and analysis

Sociodemographic data were collected using a structured form, with data on age group, race/ethnicity, place of birth, marital status, degree, and time working in prenatal care. A single open-ended interview was conducted with each participant by a scientific initiation student, a bachelor’s degree student in nursing, accompanied by a nurse professor, a doctoral student in sciences, and with previous experience in qualitative research, between January and July 2023, previously scheduled, audio-recorded, with an average duration of 45 minutes, held in a private room at the BHU itself, guided by the guiding question: what is your perception of your partner participation in prenatal care? Probing questions were also used to assist the interviews, such as how, where, and why. Thematic analysis of interviews was performed. Data purification was guided by the following stages: full transcription of interviews with the help of transkriptor software; skim reading; purification, definition of the text body, correction, and inference^(17)^. Coding was performed manually by two researchers, and a third researcher was consulted in case of uncertainty. Chart 1 shows the process of constructing the thematic categories. Participants received feedback from coding tree with the general topics that emerged from speeches (Chart 1). Analysis was carried out in light of the gender category^(12-15)^. Participants were coded with the letter N (Nurse) and with Arabic numerals assigned sequentially.

**Chart 1:** Categories and topics identified from content analysis, Brazil, 2024

Tree categories	Subcategories	Topics
**Perception of partner participation based on representations of hegemonic masculinity**	Characteristics of prenatal care for partners	Absenteeism of men in prenatal appointments
		Gateway for the inclusion of men in the healthcare network
		Weaknesses of health systems in the early recruitment of men for disease prevention
	Barriers to prenatal partner care	BHU opening hours
		Company guidelines regarding the justification for prenatal appointments by partners
		High demands on nursing professionals in BHU and the impact on the overload of professional practice
		Prioritization of maternal and child care or physiopathological aspects of pregnancy and the biomedical care model
		Gender roles, socially established and reinforced by practice agents
		Naturalization of care as an inherent characteristic of women
	Counterpoints to partner participation in prenatal care	Women’s privacy to list their doubts and even complaints regarding their partners
		Fear of fueling women’s frustrated expectations regarding partner participation in prenatal appointments
		Contrary to women’s continued treatment for syphilis during pregnancy
**Indicators of paradigm shifts regarding partner participation in prenatal care**	Reports of beneficial effects of partner participation in prenatal care	Increased maternal self-confidence
		Sharing of responsibilities for pregnancy care
		Increased family bonding
	Strategies for partner participation in prenatal care	Social awareness campaigns
		Adapting BHU opening hours
		Fostering partner integration in appointment
		Strategies to foster bonding with the pregnancy and the child

## RESULTS

Of the total, six male and five female nurses participated, belonging to the age range of 28 to 42 years. Seven were married, eight self-declared as brown and six had no children. Regarding education, seven had graduate degrees, and the majority (ten nurses) had more than one professional contract and an average of five years of prenatal care in PHC. Two thematic categories were listed based on speech coding:

### Perception of partner participation based on representations of hegemonic masculinity

In this category, participants generally characterize the representations they learn about partner participation in prenatal appointments, in which male presence was referenced as sporadic and rare. However, nurses have a positive perspective on partner participation, in the sense of trying to articulate the care network through paternity. It was also observed that men were attributed habits of not seeking healthcare services. This search is only carried out when there is a pathological condition and its symptoms established. Self-care and healthy prevention habits are neglected by men, exposing them to the possibilities of acquiring and not treating health problems.

*Well, I like to start a woman’s prenatal care with her partner. In other words, if he comes to the unit, if he has the possibility of being present during the appointment, I think it’s great! And then we have the obligation to make him feel important during the appointment. And so, just as we request tests for the pregnant woman, we also request tests for the partner. We explain to them the need for them to also take these tests. But the presence of the partner during prenatal care is still a rarity here at the unit.* (N1)

*We took advantage of this opportunity to address the health of these men, because they are far removed from healthcare services. So, it is possible to do this screening, identify these men and refer them to the doctor, depending on the need! We also assess the exams if necessary and we refer them to a doctor’s appointment, a psychologist’s appointment, as has been the case, a nutritionist’s appointment, because there are some who eat a lot, eat the wrong food, are obese, smoke, drink, and have exhausting jobs.* (N4)

*I have a husband of a pregnant woman I treat who is a longshoreman, which means he works very heavy work, carrying a lot of weight. He is hypertensive and pre-diabetic, so I have already referred him to a nutritionist and I am also monitoring him. It is a gateway to monitoring the health of these men.* (N3)

The health system operationalization does not favor the early recruitment of men to prevent the onset of more serious conditions, even when it uses strategies such as extending business hours, failing to coordinate and recruit men to PHC. This gap may be linked to models of performing the male gender, linking care practices to the female figure.

*Some time ago, the BHU started planning to extend its opening hours until later, even into the evening, to attract not only prenatal partners, but other men, for adult health, men’s health, but it didn’t go ahead. Men don’t usually come for health appointments, only when they already have an illness. If they don’t have any symptoms, they don’t seek the service.* (N4)

Participants reported some social elements that they understand as barriers to effective male participation in prenatal care, such as guidelines of Brazilian companies regarding justifications for men’s absence from work to participate in prenatal appointments, such as the requirement for a medical certificate and, also intertwined with these guidelines, BHU opening hours.

*We work during business hours. And most of the time, men are working during these hours. When they are self-employed, they can make this participation viable, but when they are employed under the CLT regime, it is more difficult for them to miss work and accompany their wife or baby to the appointment, even with the possibility of us providing a certificate of attendance, which is another problem as well.* (N5)

*Some large companies where these men work do not accept a certificate of attendance; they want a doctor’s certificate. And so, since it is not an illness, they are not sick, so it is not a doctor’s certificate. We already need to explain to two HR managers here at the unit the difference between a doctor’s certificate and a certificate of attendance.* (N1)

The high demand for activities by nursing professionals in BHU and the impact of the overload of yet another task in professional practice were verified. Related to this overload, it is inferred that maternal and child care is prioritized, which can also be understood as an emphasis on pregnancy pathophysiological aspects and the biomedical care model. The socially established gender roles are reinforced by agents of practice, such as the naturalization of care as an inherent characteristic of women or the attribution of the characteristic of participatory fathers to men who delegate the care of children to the figure of a woman, even if she is not the child’s mother.

*Well, in my case, I have this flaw of not really guiding my partner regarding prenatal care. We have a very high demand for care for women, who end up being prioritized, perhaps due to the shortage of human resources.* (N2)

*Women are more concerned with scheduling appointments and exams. When their partners come, they are the ones who schedule exams and get medicine. Women take care of their husbands, because women are more interested in taking care of people, the house, the family, and seeing that everyone is well, right?* (N6)

*When it comes to high-risk prenatal care, they get very desperate, more desperate than the women themselves, but also because there is a lack of information, they are people who are very lacking in education, but when they understand, they calm down. But it is usually the woman who calms them down* [laughs]. (N7)

*Just now I saw a father. When the child cried, he would go to his grandmother. An 11-day-old baby. He is a very involved father, but he has to go to his grandmother to make the child stop crying.* (N8)

Participants listed elements that they considered counterpoints to partner participation in prenatal appointments, such as the reduction of women’s privacy to list their doubts and also complaints, sometimes related to their own relationship with their partners. Care should be taken so that the request for partner participation and guidance on the benefits of this participation do not generate frustrated expectations in pregnant women, being another element of emotional overload for women. Participants report that, in some situations, such as cases of syphilis during pregnancy, the attempt to recruit partners for joint treatment with women ends up in the women themselves not attending appointments.

*Sometimes, they become withdrawn when their partner comes. When they don’t come, they become more relaxed, right? Even to complain about them and to talk more intimately.* (N8)

*We feel sad because we know that when a woman is pregnant, she is in a very vulnerable state, she is much more sensitive. So, we try as much as possible not to make her feel even more apprehensive about her partner not wanting to come, right? I always try to say this, when I ask about them and they say they are at home, they didn’t want to come because they were sleeping and the appointment was taking too long: look, don’t worry about it, it’s normal and it always happens that the partner doesn’t come.* (N9)

*There are many cases of syphilis here, a lot indeed. So, it is important to get the partner treated in these cases, but it is also very difficult. Sometimes, even the woman ends up not coming anymore.* (N11)

### Indicators of paradigm shifts regarding partner participation in prenatal care

Participants reported beneficial effects that they observe in their professional practice, including increased maternal self-confidence, sharing of responsibilities for pregnancy care and, therefore, reducing the burden on women and increasing paternal bond with the child and family.

*Partner participation even gives the mother more confidence, especially when she is a first-time mother, when she is very young. And here we have a lot of very young mothers who arrive very nervous and anxious, so coming with a partner gives them a sense of security. We explain everything to both of them, give them the right instructions. Then they also call their attention to it, because there are many mothers who avoid getting vaccinated, who don’t take their medicines properly, and they are there supervising and helping them.* (N5)

*I have a pregnant woman who doesn’t understand anything I explain, but then I explained it to her partner, and then he, in his own way, explained it to her and she understood, that usually happens a lot too.* (N10)

*I have an example here: a father who, when he comes and I put on the sonar to listen to the baby, sometimes, I can’t find it. Then the father goes and talks to the baby, and then I can hear the baby’s heartbeat. The mother even said that the baby can’t hear his voice because, when she hears it, it moves.* (N6)

Nursing professionals use some strategies to reach partnerships for prenatal care to enable care for the target audience of the strategy. Among them are an information campaign on the importance of paternal presence during pregnancy for a healthy child development and adjustment of schedules, whether offering appointments at night or during lunch hours. Another strategy was man integration in appointments, favoring their participation in procedures that establish a link with the pregnancy and the child, such as fetal heart rate auscultation (FHR).

*I think there needs to be a major awareness campaign for the community, for society, about the importance of this work that men do with pregnant women, right? The baby’s father.* (N4)

*One strategy we use is to adapt the schedule. For example, if they are free at 11:30 for lunch, they even let us know and end up sacrificing their lunch time to be here at the appointment time. But this is complicated for them, and it ends up being complicated for us too, because it is difficult for us to work with a schedule that has a schedule, right? But as far as possible, this is a strategy that has worked.* (N1)

*They like to be present when we do the FHR auscultation, and we have to take the opportunity to explain to them that every time they come for an appointment, we will do the FHR auscultation. And then we take the opportunity to say that the baby hears the father’s voice, that the baby perceives the father’s reactions. So, the father feels like he is participating in this process.* (N4)

## DISCUSSION

This study identified absenteeism among men during prenatal care, expressing gender relations. Low male awareness of care was evidenced, in addition to barriers to access to healthcare services due to working hours and rigid labor issues. Professional overload and structural limitations contributed to a greater emphasis on maternal and child care. The presence of partners during prenatal care brought to light manifestations of hegemonic masculinities during care, with the need for nurses to manage situations to promote privacy and comfort for women and man involvement in child care. However, benefits of prenatal care for men were evidenced, from nurses’ perspective, with increased bonding and trust with women, co-responsibility in care, development of health education activities focusing on care during pregnancy, labor and birth, puerperium and newborn care.

Cultural representations anchored in men as providers, who are rarely present in conception care due to their busy schedules, are in line with the findings of this study, in which the low man participation in prenatal care was also attributed to time constraints related to work^(18)^, institutional challenges of companies and health units, inadequate structures, and healthcare professionals with inadequate training and preparation to deal with this public. This perspective reproduces the idea that care is a female function and represents a barrier to changing gender norms. Also noteworthy is the unclear role of how important men believe it is to be involved in the prenatal period, sociocultural factors regarding gender roles, and the perception that healthcare service messages are aimed at women^(19)^.

A study carried out in Iran found that men have little or no knowledge about the physical changes that occur during pregnancy and childbirth, adequate nutrition, recommended physical activities, sexuality, signs of risk, mental and psychological changes during pregnancy, childbirth, the puerperium and neonatal care^(20)^, which highlights the need for their participation in appointments with nurses and healthcare professionals with notable experience in health education to obtain guidance that gives them self-confidence in care^(21)^, and can go against hegemonic representations of masculinity and paternity. The current male profile is unprepared and insecure about the expectations that must be met by the father^(22)^. This is due, in part, to the discontent and exclusion coming from some agents of practice who, when providing prenatal care, ignore the presence of partners during appointments, focusing guidance only on pregnant women, leaving the fathers in the background^(23)^.

In this regard, a prospective cohort study conducted in England explored the opinions and experiences of 42 parents regarding perinatal mental health after the birth of their first child, and found that they experienced psychological distress but were reluctant to express their needs for support^(24)^. An integrative review also found that depressed parents may experience strong feelings of worthlessness, guilt, and less confidence. This condition may influence parents’ response to pregnancy, particularly their ability to bond with the fetus. Depression also inhibited the development of a positive bond, as parents tended to have negative thoughts and emotions regarding the fetus^(25)^. These results point to the importance of mental healthcare needs of both partners being identified and managed by healthcare professionals, with a view to the family’s psychological well-being.

Among the barriers described for reaching partners during the prenatal period, a study conducted in municipalities in the Brazilian Amazon found that most pregnant women are unaware of their partner’s prenatal strategy and claim that they were not informed by nurses about their partners’ presence at appointments^(22)^. On the other hand, in line with some of the findings of this research, such as women choosing not to have their husbands attend appointments, a study conducted in Ethiopia found that some women mentioned that the presence of their husbands in health units makes it difficult for them to express their feelings or opinions freely and honestly. Thus, women often did not tell their husbands when healthcare professionals requested their presence during the subsequent prenatal appointment. This conditioned partner participation to the level of relationship between the couples and professionals’ knowledge about the emotional dynamics of the couples being treated^(26)^.

Existing biomedical model rarely includes information aimed at men during prenatal appointments or on psychological and social aspects. The lack of education and content aimed at men can leave many fathers with unanswered questions and feeling excluded from the process^(22,23)^. Thus, professional practices demonstrate the persistence of biologicist and fragmented practices, forged from perceptions that reiterate gender subordination, accompanied by failure to recognize the responsibility of the area for addressing social problems.

It is worth noting that the schemes of the sexualized unconscious are the result of historical structures, highly differentiated and born from a social space, which are reproduced through learning linked to the experience that agents - mainly and especially those of practice - have of the structures of the space. This means that the unconscious takes root and reproduces itself^(15)^. Coping with the model of sexualized unconscious, based on hegemonic masculinity, demands its recognition by health and related areas, and requires overcoming fragmented practices that disregard the comprehensiveness between biological and social as well as the leading role of nursing as an agent of transformation of objective reality. Emancipatory education emerges as a possibility for developing intersectoral coping strategies based on the collective construction of knowledge and interventions guided by gender equality.

Male nurses often challenge traditional stereotypes of masculinity through their professional choices and behavior in the workplace^(27,28)^. These professionals are trained to be caring, empathetic, and attentive to the needs of individuals, families, and the community, characteristics often associated with female care in traditional society^(27)^. Thus, these professionals are expected to present a more flexible and progressive view of masculinity that incorporates aspects of care and emotional sensitivity, which can be positive examples for the population.

In contrast, masculinity among men in the territory who do or do not accompany their partners to prenatal appointments can vary widely, influenced by cultural, social and economic factors. The traditional view of masculinity, in which men are seen as the providers and women as the primary caregivers, still prevails. In this view, healthcare, especially related to maternity, is often considered a female responsibility^(2)^. Man participation in prenatal care has the potential to contribute to the deconstruction and disruption of traditional and patriarchal norms, demonstrating a commitment to a more inclusive and participatory masculinity.

In this study, it was possible to observe the report of absenteeism in appointments by partners. It is possible to verify that, even with the perception of the man’s desire to participate in the process of pregnancy and birth of their child^(29)^, in Brazil, there are still cultural and institutional barriers that prevent them from exercising their right, such as labor guidelines and legislation and preparation of practical agents in the assistance at the entrance of services. Article 473 of Law 13,257/2016 stated that employees could not attend work without loss of salary for up to two days to attend medical appointments and complementary exams during the pregnancy of their wife or partner.

However, it is worth noting that this article of law, published in the same year as the publication of the Partner Prenatal Strategy^(8)^, became an impediment, therefore paradoxical, to partner participation in prenatal appointments, since the Brazilian Ministry of Health itself recommends a minimum of six prenatal appointments as a model of adequate care. Only in 2022, a provisional measure (PM) extended the interval to up to six days of monitoring. This PM was included and can certainly be understood in a sociocultural context of overcoming hegemonic masculinity and paternity and, also, as an incentive for greater man participation in the family structure and in care tasks^(30)^. The link between health and the creation of labor laws that promote healthy parenting must be strengthened, with the possibility of exchange between care functions^(2)^.

Some elements highlighted in the literature are listed as being associated with greater partner participation in prenatal care, such as invitation by the partner, flexible hours, overestimating the risk of transmission of HIV and other sexually transmitted infections (STIs) from mother to child^(18)^, accepting to perform rapid tests to protect partners and fetuses during this vulnerable period, and integrating men into the appointment^(11,18)^. A mixed-method study conducted in Ethiopia demonstrated that working in cooperation with community leaders can be a good strategy to influence men’s behavior, and they are also a source of information for policy makers on strategies to overcome barriers to positive maternal health outcomes, especially at the community level^(31,32)^.

In the United States, it was observed that using applications aimed at introducing men to prenatal education was seen as positive. Participants recommended the addition of interactive modules, such as a financial planning tool and videos, to strengthen the application. Men were open to information on how to share with their partners the care of a healthy pregnancy and promote the health of their unborn children^(21)^. Home visits, with partner integration, can also be used as a strategic tool. Healthcare professionals should care for pregnant women in the presence of their partners. It is also recommended that couples receive training together during the gestational period and the postpartum period for maternal and neonatal care^(32)^. Fathers’ experiences are affected and there is an increase in confidence when they take over and deal with the responsibilities of care in the first days at home^(6)^. These strategies can help with the weaknesses in recruiting men observed in the results of this research.

In addition to the strategies outlined, it is important to ensure that not only actions are created to foster bonds between adults and children, nor only man involvement as instrumental in the care of women and children and the opportunity for them to undergo routine preventive examinations, but, above all, that identities can be constructed that are articulated with the self-realization of human beings^(2)^. The literature infers that paternal identity construction involves a complex subjective, conscious and unconscious work of elaborating the inheritances received from one’s own parents and transforming them by providing care to children, overcoming the models of representation of paternity and masculinity perpetrated over generations and redefining paternity^(4,22)^.

In order to break a certain paradigm, based on a partner who participates little in raising children and who neglects self-care, the Brazilian State must provide means to bring a new representation of masculinity and paternity by anchoring a perspective of active parenthood and shared care from the pre-conception period, bringing men into health units, where they must be welcomed and involved in the care processes, favoring the construction of paternity and the formulation of healthy family bonds.

### Study limitations

This study has some limitations. The first is that it was conducted only with the professional category of nurses, which does not provide a clearer and more cohesive view of the perception of agents of health practices that make up the Brazilian State. The second is that the responses led the study to a heteronormative perspective. Thus, discussions about preconception, conception and parenting aspects among same-sex couples were dispelled. However, in this regard, the State policy itself establishes partners as the target recipient of care, dispelling the different forms of partnerships involved in the parenting process. Moreover, it is important to be cautious so as not to treat the representations expressed here in fixed ways, as they are fluid to continuous social changes. It is also worth noting that nursing, as a professional category, encompasses a group of individuals who differ in their ways of seeing life, as they have distinct individual experiences and cannot be compartmentalized in a rigid way within a single sphere of their lives, in this case, the professional category to which they belong.

### Contributions to health, nursing or public policy

Campaigns to inform about the importance of partners’ presence during pregnancy for a healthy child development should be encouraged and financed by state public policies. Studies on the feasibility of extending opening hours of BHU during non-business hours should be conducted.

The curricular components aimed at training nurses must address the harmful issues of hegemonic masculinity for society as a whole. Primarily, the construction of parental identities of care articulated with the self-realization of human beings must be promoted.

Additionally, implementing support programs specifically for men, such as fathers’ groups or counseling sessions, can help address the concerns and insecurities that many men may have about their role during pregnancy. These programs can provide practical and emotional information, helping to normalize male participation in prenatal care.

Incorporating these reflections on masculinity into nursing practices not only improves prenatal care quality, but also contributes to building a more equitable and inclusive society. By actively involving men in care, nurses can promote a culture of health where all family members are valued and supported in their needs and contribute to achieving the SDGs.

## FINAL CONSIDERATIONS

Despite the recognition by professionals of the importance of prenatal care for men, gender issues persist in the daily care provided in the community. Prenatal care was strongly represented by markers of hegemonic masculinity, characterized by partner absenteeism, motivated by macro and micro issues related to paternity construction, such as the difficulty of justifying men’s absence from prenatal appointments in work services and, in the domestic sphere, partners choosing not to attend or, even attending, but outsourcing the care of children to women even during appointments, such as grandmothers. Gender roles were reinforced by professionals in practice, and care was naturalized as inherent to female roles. The negative aspects related to partner participation were also intertwined with representations of hegemonic masculinity, such as women’s lack of privacy to complain about their partners to healthcare professionals. Nurses’ workload in PHC was intertwined with prioritization of maternal and child pathophysiological aspects to the detriment of the biopsychosocial benefits of welcoming and fostering partners’ comprehensiveness in care. Furthermore, the teams’ service hours, as well as the harsh working conditions, remain challenges. These elements are related to the conceptual dimensions of professional training, aimed at meeting the production of demands with a focus on biological perspectives and also represented in models of hegemonic masculinity and femininity. Thus, there are gaps in the strategies for identifying and adhering to men’s health and, consequently, in the construction of a welcoming masculinity, paternity and parenthood with the driving force to carry out structured transformations.
