# Supplementary material for: Nurses’ representations about partner participation in prenatal care: perspective on masculinities
Source: Rev Bras Enferm. 2025 Mar 14;78(Suppl 1):e20240160. doi: 10.1590/0034-7167-2024-0160 (PMC11913134; doi:10.1590/0034-7167-2024-0160)
Supplement: Translation

# Translation: Representações de enfermeiros sobre a participação de parceiros no cuidado pré-natal: perspectiva sobre as masculinidades

**DOI:** 10.1590/0034-7167-2024-0160pt

**Authors:** Lise Maria Carvalho Mendes, Nayara Gonçalves Barbosa, Edson Silva do Nascimento, Ana Karina Bezerra Pinheiro, Flávia Azevedo Gomes-Sponholz

**Affiliations:** I Universidade Federal do Amapá. Macapá, Amapá, Brasil; II Universidade de São Paulo. Ribeirão Preto, São Paulo, Brasil; III Universidade de São Paulo. São Paulo, São Paulo, Brasil; IV Universidade Federal do Ceará. Fortaleza, Ceará, Brasil

**Keywords:** Cuidado Pré-Natal, Paternidade, Saúde do Homem, Educação Pré-Natal, Perspectiva de Gênero

## Abstract

**Objetivos:**

compreender as representações de enfermeiros sobre a participação de parceiros na assistência pré-natal.

**Métodos:**

estudo exploratório, descritivo, qualitativo, realizado em quatro Unidades Básicas de Saúde, situadas em dois municípios da região da Amazônia brasileira. Foram realizadas entrevistas abertas com 11 enfermeiros atuantes na assistência pré-natal há, pelo menos, seis meses, entre janeiro e julho de 2023. Foram excluídos profissionais que estivessem sob licença saúde ou férias. A organização dos dados ocorreu mediante análise de conteúdo, e a análise teórica foi embasada na categoria gênero.

**Resultados:**

foram observadas percepções da participação do parceiro alicerçadas em representações da masculinidade hegemônica, mas, também, observaram-se indicadores de mudança de paradigma referentes à paternidade.

**Considerações Finais:**

os profissionais reforçam os papéis de gênero tradicionais, tratando o cuidado como algo inerente às mulheres. Foi observado também que a sobrecarga da categoria leva à perpetuação de modelos biomédicos de cuidado.

## INTRODUÇÃO

As relações paternas na família contemporânea têm apresentado significativas transformações, principalmente após a década de 1970, com o advento de acesso a métodos contraceptivos e inserção em massa das mulheres no ambiente de trabalho externo ao lar, somado, também, à disseminação de estudos feministas acerca dos papéis de gênero^(1)^. Essas transformações impulsionaram o surgimento de uma nova expressão do papel masculino na sociedade^(2)^. A expressão e a necessidade desses novos modelos de expressar o papel masculino emergem também das evidências dos resultados negativos referentes ao modelo de masculinidade hegemônica que vigorava anteriormente e que reverbera nas representações dos papéis assumidos por homens no cuidado de si e nos modelos de paternidade vigentes^(3)^.

Os modelos de masculinidade tradicionais e a maneira como se dá a socialização masculina fragilizam ou mesmo afastam os homens das preocupações com o autocuidado e a busca pelos serviços de saúde^(2-4)^. A negação do medo, a exposição ao risco e o silenciamento acerca das dores físicas e psíquicas são considerados traços da masculinidade hegemônica, valorizados por estarem diametralmente opostos à fragilidade e emotividade características da feminilidade hegemônica. Tais elementos justificam a pouca procura masculina pelos serviços de Atenção Primária à Saúde (APS), os elevados índices de morbimortalidade por doenças evitáveis ou envolvimento em situações de violência^(2,3)^. A perspectiva do cuidado pode seguir um caminho positivo, quando incorpora a ideia de que ao homem também é permitida uma atenção consigo próprio^(4)^.

As transformações dos papéis de gênero na sociedade podem gerar benefícios a curto, médio e longo prazo, por isso apresentam-se como importante ao debate científico. Entre estes benefícios, observa-se que mulheres acompanhadas na rotina de pré-natal por seus parceiros apresentam menores complicações durante o trabalho de parto, parto e pós-parto, e melhora de aspectos emocionais durante a gestação^(5)^. Metanálise verificou que o favorecimento da paternidade ativa, através do compartilhamento de responsabilidades desde as consultas pré-concepcionais até os cuidados com a criança, reverbera no vínculo formado com a criança, repercutindo de forma positiva em aspectos biopsicossociais e melhorando os resultados para toda a família em países de baixa e média renda^(6)^. Verificou-se, dessa forma, a necessidade de uma mudança de paradigma, para que se alcancem melhores indicadores de saúde, vínculos familiares fortalecidos e uma sociedade menos conflituosa^(3)^.

O Brasil foi pioneiro na América Latina ao criar uma política pública destinada à saúde dos homens, das quais um de seus objetivos foi o de ampliar o acesso e o acolhimento aos homens nos serviços e programas de saúde, através da inclusão do tema da paternidade e do cuidado^(7)^. As ações voltadas ao planejamento reprodutivo como uma estratégia essencial para qualificar a atenção à gestação, ao parto e ao nascimento, com a inclusão dos parceiros, tomaram destaque em 2016, com a formulação da Estratégia Pré-Natal do Parceiro. Esta estratégia constitui-se uma porta de entrada para os homens na rede de serviços de saúde, aproveitando sua presença nas consultas de pré-natal para ofertar exames de rotina e atividades educativas, buscando a integralidade à rede de cuidado para esta população^(8)^.

Destaca-se que uma maior atenção à temática poderia contribuir para o alcance dos Objetivos de Desenvolvimento Sustentável (ODS) da agenda 2030, principalmente o terceiro, relacionado à saúde e bem-estar, com ênfase às metas de redução das mortes prematuras por doenças crônicas não transmissíveis (DCNTs), mortalidade materna, mortalidade infantil, abuso de substâncias, assegurar o acesso universal aos serviços de saúde sexual e reprodutiva, bem como o quinto, que visa à redução das iniquidades de gênero^(9)^. A participação do homem nas consultas pré-natais propicia um momento oportuno que pode auxiliar no processo de construção de novas representações referentes à paternidade e a novos modelos de masculinidades, aproximando os homens aos serviços de saúde e às práticas de cuidado^(8)^.

No entanto, a literatura aponta alguns fatores como impeditivos à participação dos homens às consultas de pré-natal, como questões relacionadas aos papéis de gênero, o horário de funcionamento das Unidades Básicas de Saúde (UBS), a escassez de ações educativas voltadas aos parceiros e a ausência de incentivo por parte das equipes de saúde à participação dos parceiros durante as consultas e realização de exames^(10)^. Traçar estratégias e criar ferramentas para captar a presença dos parceiros nas consultas pré-natais e no ambiente de cuidado tem sido um desafio mundial^(6,11)^.

Ressalta-se que a equipe de enfermagem possui um papel categórico na luta pela integração de homens durante o ciclo gravídico-puerperal como parte do cuidado de enfermagem e, consequentemente, como forma de promover a integralidade do cuidado, através do acolhimento, da escuta qualificada e do incentivo à conexão dos parceiros com a parentalidade sadia. Devido à relevância da temática, faz-se imperiosa a abordagem das representações de enfermeiros sobre a inclusão e participação dos parceiros nas consultas de pré-natal, visto que a enfermagem, enquanto ciência, é formuladora de estratégias que viabilizam a integração e promovam, sobretudo, o aprimoramento do cuidado.

## OBJETIVOS

Compreender as representações dos enfermeiros sobre a participação dos parceiros na assistência pré-natal.

## MÉTODOS

### Aspectos éticos

Este estudo respeitou as exigências nacionais e internacionais regulamentadoras de pesquisas envolvendo seres humanos, de acordo com a Resolução nº 510/2016 do Conselho Nacional de Saúde (CNS). Obteve parecer favorável em dezembro de 2022 pelo Comitê de Ética em Pesquisa de uma universidade pública do extremo norte do Brasil. Os participantes manifestaram consentimento à participação do estudo mediante assinatura do Termo de Consentimento Livre e Esclarecido (TCLE). As entrevistas foram codificadas através da letra E (enfermeiro), procedidas por um número ordinal. Foram utilizadas, como cumprimento ao rigor metodológico, as diretrizes do *COnsolidated criteria for REporting Qualitative research* (COREQ).

### Tipo de estudo e referencial teórico-metodológico

Estudo exploratório, descritivo, de abordagem qualitativa, do tipo pesquisa social estratégica, no qual utilizou-se a categoria gênero como lente analítica. Esta categoria, em primeiro plano, centra-se na compreensão de que há distinção do que seja sexo biológico e do que seja sexo social. O primeiro refere-se às diferenças anátomo-fisiológicas, portanto, biológicas, existentes entre os homens e as mulheres, e o segundo diz respeito à expressão que assumem essas diferenças do sexo biológico nas distintas sociedades^(12)^. Dessa forma, gênero pode ser compreendido como o elemento constitutivo das relações sociais baseado nas diferenças percebidas entre os sexos^(13)^.

Sobre esse aspecto, destaca-se que há diversas formas de performar os papéis atribuídos aos homens, ou seja, a masculinidade, que variam de acordo com os aspectos culturais e as interseccionalidades de classe, raça e geração^(4)^, especificidades estruturantes entre homens e mulheres pelas diferenças de exigências que recaem sobre cada papel. Entre as diferentes formas de expressar a masculinidade, a predominante é aquela que se erige da cultura patriarcal a qual julga aos homens superioridade às mulheres^(14)^. A validação deste tipo de masculinidade circunscreve-se em características que devem estar presentes em um homem, como ser assertivo, não demonstrar sentimentos, envolver-se em condutas de risco, relacionar-se sexualmente com o maior número de mulheres possível, não envolver-se em atividades de cuidado consigo, com a prole e com o lar, entre outros aspectos nocivos à saúde biopsicossocial^(4,14)^.

Assim como nas masculinidades, há distintas formas de se exercer a paternidade, não sendo a sua abordagem um assunto universalmente determinado^(4)^. Reconhece-se que essa determinação histórica e socialmente construída influencia a percepção, a visibilidade, as práticas e o enfrentamento dos modelos de masculinidade e paternidade dominantes. O inconsciente que governa as relações entre os sexos, tanto em sua origem individual quanto em sua evolução coletiva, está profundamente ligado a um conjunto de campos e agentes que competem pelo monopólio da definição legítima das práticas e dos discursos sexuais, impondo essas definições^(15)^. Esse inconsciente representacional está disperso na sociedade e pode estar presente também nos discursos e/ou práticas dos agentes de cuidado do Estado. Portanto, considera-se que a categoria social de gênero pode determinar a construção histórica e social das representações presentes nos discursos sobre a participação do parceiro na assistência pré-natal pelos enfermeiros.

### Cenário do estudo e fonte de dados

A pesquisa foi realizada em quatro UBS situadas em dois municípios do extremo-norte da Amazônia brasileira. Optou-se pelas perspectivas metodológicas dispostas por Bourdieu, pela não identificação dos municípios de coleta, para que não haja possibilidade de identificação dos participantes e, desse modo, proteger e assegurar as confidências recolhidas em uma relação de confiança, e abrandá-las dos constrangimentos aos quais suas palavras poderiam ser expostas^(16)^. Os participantes do estudo foram 11 enfermeiros atuantes na assistência pré-natal há, pelo menos, seis meses. Foram excluídos profissionais que estivessem sob licença saúde ou férias. A amostra foi intencional. Desta forma, as UBS foram escolhidas por serem unidades estratégicas nos municípios atuantes, e todos os 14 enfermeiros dessas UBS foram convidados a participar do estudo. Um enfermeiro estava de licença-maternidade no período, e outros dois recusaram-se a participar do estudo, mediante a sobrecarga de tarefas e incompatibilidade de agenda.

### Coleta, organização e análise dos dados

Os dados sociodemográficos foram coletados por meio de um formulário estruturado, com dados a respeito da faixa etária, cor/etnia, naturalidade, estado civil, titulação e tempo de atuação na assistência pré-natal. Foi realizada uma única entrevista aberta com cada participante por uma aluna de iniciação científica, acadêmica do curso de bacharelado em enfermagem, acompanhada por docente enfermeira, doutoranda em ciências e com experiência pregressa em pesquisa qualitativa, entre janeiro e julho de 2023, agendada previamente, áudio-gravada, com média de 45 minutos de duração, realizada em sala privativa nas próprias UBS, guiada pela pergunta norteadora: qual a sua percepção sobre a participação do parceiro na assistência pré-natal? Foram utilizadas ainda perguntas de sondagem como auxílio às entrevistas, a exemplo de como, onde e por quê. Foi realizada a análise temática das entrevistas. O depuramento dos dados foi orientado pelas seguintes etapas: transcrição na íntegra das entrevistas com auxílio do *software transkriptor*; leitura flutuante; depuração definição do corpo textual, correção e inferência^(17)^. A codificação foi realizada manualmente por duas pesquisadoras, e uma terceira pesquisadora foi consultada em caso de dubiedade. O processo de construção das categorias temáticas está disposto no Quadro 1. Os participantes receberam uma devolutiva da árvore de codificação com os temas gerais que emergiram dos discursos (Quadro 1). A análise ocorreu à luz da categoria de gênero^(12-15)^. Os participantes foram codificados com a letra E (Enfermeiro) e com números arábicos atribuídos sequencialmente.

**Quadro 1:** Categorias e temas identificados a partir da análise de conteúdo, Brasil, 2024

Categorias árvore	Subcategorias	Temas
**Percepção da participação do parceiro alicerçadas em representações da masculinidade hegemônica**	Características da assistência pré-natal ao parceiro	Absenteísmo de homens nas consultas de pré-natal
		Porta de entrada para inserção do homem na rede de assistências
		Fragilidades dos sistemas de saúde na captação precoce de homens para prevenção de agravos
	Barreiras para assistência pré-natal ao parceiro	Horário de funcionamento das UBS
		Diretrizes de empresas diante da justificativa de comparecimento às consultas de pré-natal pelo parceiro
		Elevadas demandas dos profissionais enfermeiros nas UBS e o impacto na sobrecarga da prática profissional
		Priorização da assistência materno-infantil ou dos aspectos fisiopatológicos da gestação e modelo biomédico assistencial
		Papéis de gênero, estabelecidos socialmente e reforçados pelos agentes da prática
		Naturalização do cuidado como característica inerente à mulher
	Contrapontos da participação do parceiro na assistência pré-natal	Privacidade da mulher para elencar suas dúvidas e, inclusive, queixas referentes ao parceiro
		Receio de alimentar frustrações de expectativas nas mulheres pela participação dos parceiros nas consultas pré-natais
		Efeito contrário para continuidade de tratamento da sífilis na gestação pela mulher
**Indicadores de mudanças de paradigmas referentes à participação do parceiro no pré-natal**	Relatos de efeitos benéficos na participação do parceiro no pré-natal	Aumento da autoconfiança materna
		Partilha de responsabilidades com o cuidado gestacional
		Aumento do vínculo familiar
	Estratégias para participação do parceiro no pré-natal	Campanhas de conscientização social
		Adequação dos horários das UBS
		Fomento à integração do parceiro na consulta
		Estratégias de fomento do vínculo com a gestação e a criança

## RESULTADOS

Do total, participaram seis enfermeiros do sexo masculino e cinco do sexo feminino, pertencentes à faixa etária de 28 até 42 anos. Sete eram casados, oito autodeclararam-se pardos e seis não tinham filhos. Em relação à formação, sete possuíam pós-graduação, e em sua maioria, (dez enfermeiros), com mais de um vínculo profissional e média de tempo de assistência pré-natal na APS de cinco anos. Foram elencadas duas categorias temáticas a partir das codificações dos discursos:

### Percepção da participação do parceiro alicerçadas em representações da masculinidade hegemônica

Nessa categoria, os participantes caracterizam de forma geral as representações que apreendem sobre a participação dos parceiros nas consultas de pré-natal, em que a presença masculina foi referenciada como esporádica e rara. No entanto, os enfermeiros têm a perspectiva positiva da participação dos parceiros, no sentido de tentar articular a rede de cuidados através da paternidade. Observou-se também que aos homens foram atribuídos hábitos de não buscarem o serviço de saúde. Esta busca só é realizada quando há um quadro patológico e sua sintomatologia instaurados. O autocuidado e os hábitos saudáveis de prevenção são negligenciados pelos homens, expondo-os às possibilidades de adquirir e de não tratar os agravos de saúde.

*Bom, eu gosto de começar o pré-natal da mulher junto com o parceiro dela. Ou seja, se ele vem pra unidade, se ele tem a possibilidade de no horário do atendimento estar presente, acho ótimo! E aí nós temos a obrigação de fazer com que ele se sinta importante na consulta. E assim, como a gente pede os exames pra gestante, se pede também os exames para o parceiro. Explica pra eles a necessidade de eles também realizarem esses exames. Mas a presença do parceiro no pré-natal ainda é uma raridade aqui na unidade.* (E1)

*Aproveitamos o momento para tratar a saúde desses homens, porque eles são muito afastados dos serviços de saúde. Então, é possível fazer esse rastreamento, pegar esse homem e já encaminhar pro* médico*, dependendo da necessidade! A gente avalia os exames também se for preciso e a gente encaminha pra consulta com médico, consulta com psicólogo, como tem acontecido, consulta com nutricionista, porque tem alguns que comem muito, comem errado, estão obesos, são fumantes, bebem, além de terem um trabalho exaustivo.* (E4)

*Eu tenho um marido de uma grávida que atendo que ele é estivador, ou seja, ele trabalha com serviço muito pesado, ele trabalha carregando muito peso. E ele é hipertenso, ele está pré-diabético, e aí eu já passei pra nutricionista e vou acompanhando também. É uma porta de entrada para rastreamento da saúde destes homens.* (E3)

A operacionalização do sistema de saúde não favorece a captação precoce de homens para prevenção de instauração de quadros mais graves, mesmo quando lança mão de estratégias como a ampliação de horário de atendimento, não conseguindo articular e captar os homens à APS. Esta lacuna pode estar articulada aos modelos de performar o gênero masculino, articulando as práticas de cuidado à figura do feminino.

*Há um tempo atrás, a UBS começou um planejamento de estender o horário de atendimento até mais tarde, até à noite mesmo, para captar não só os parceiros de pré-natal, mas outros homens, para saúde do adulto, a saúde do homem, mas não foi pra frente. O homem não tem costume de vir pra consulta de saúde, só quando já tem uma doença. Se não tem um sintoma, ele não procura o serviço.* (E4)

Os participantes relataram alguns elementos sociais que compreendem como barreira para efetivação da participação do homem na assistência pré-natal, como as diretrizes de empresas brasileiras diante das justificativas de falta ao serviço para participação de consulta pré-natal pelo homem, a exemplo da exigência de atestado médico e, imbricado também a essas diretrizes, o horário comercial de funcionamento das UBS.

*Nós trabalhamos em horário comercial. E os homens, na maioria das vezes, nesse horário, estão trabalhando. Quando eles têm um serviço autônomo, até que conseguem viabilizar essa participação, mas quando ele é empregado em regime de CLT, é mais difícil pra ele faltar ao trabalho e acompanhar a esposa ou o bebê no atendimento, mesmo com a possibilidade da gente entregar um atestado de comparecimento, que é um outro problema também.* (E5)

*Algumas empresas grandes em que esses homens trabalham não aceitam atestado de comparecimento; eles querem um atestado médico. E, assim, como não é uma doença, eles não estão doentes, então não é um atestado médico. Nós já precisamos aqui na unidade estar explicando pra duas gerentes de RH a diferença entre atestado médico e o atestado de comparecimento.* (E1)

A elevada demanda de atividades dos profissionais enfermeiros nas UBS e o impacto da sobrecarga de mais uma atribuição na prática profissional foram verificados. Relacionado a esta sobrecarga, infere-se a priorização da assistência materno-infantil, que também pode ser compreendida como ênfase aos aspectos fisiopatológicos da gestação e ao modelo biomédico assistencial. Os papéis de gênero estabelecidos socialmente são reforçados pelos agentes da prática, a exemplo da naturalização do cuidado como característica inerente ao feminino ou de atribuir à característica de pai participativo ao homem que delega o cuidado da criança à figura de uma mulher, mesmo que não seja a mãe da criança.

*Bom, no meu caso, eu tenho essa falha de realmente não orientar o parceiro quanto ao acompanhamento do pré-natal. Nós temos uma demanda muito elevada de atendimento às mulheres, que acabam sendo priorizadas, talvez pela escassez de recursos humanos.* (E2)

*As mulheres são mais preocupadas em agendar consultas, exames. Quando os parceiros vêm, são elas que vão marcar exames, pegar remédio. As mulheres cuidam dos maridos, porque mulher tem mais isso de gostar de cuidar das pessoas, da casa, da família, de ver todo mundo bem, né?* (E6)

*Quando se trata de pré-natal de alto risco, eles ficam muito desesperados, são mais desesperados que as próprias mulheres, mas também porque falta muita informação, são pessoas muito carentes de estudo, mas, quando eles compreendem, ficam calmos. Mas é geralmente a mulher que acalma eles* [risos]. (E7)

*Agora mesmo eu atendi um pai. Na hora que a criança chorava, ele já procurava a vó. Um bebezinho de 11 dias. Ele é um pai bem participativo, mas tem isso de procurar a vó pra fazer a criança parar de chorar.* (E8)

Os participantes elencaram elementos que consideraram contrapontos à participação do parceiro nas consultas de pré-natal, como a diminuição da privacidade da mulher para elencar suas dúvidas e, também, queixas, algumas vezes, referentes à própria convivência com o parceiro. Deve-se ter cautela para que a solicitação de participação do parceiro e as orientações sobre o benefício desta participação não gerem expectativas frustradas nas gestantes, sendo mais um elemento de sobrecarga emocional às mulheres. Os participantes referem que, em algumas situações, a exemplo dos casos de sífilis na gestação, a tentativa de captação do parceiro para tratamento conjunto à mulher culmina no absenteísmo da própria mulher às consultas.

Às vezes*, elas ficam retraídas quando o parceiro vem. Quando eles não vêm, elas já ficam mais soltas, né? Até pra reclamar deles e pra conversar de forma mais íntima.* (E8)

*A gente fica triste, porque a gente sabe que, quando a mulher está gestante, ela está num estado de muita vulnerabilidade, ela está muito mais sensível. Então, a gente procura ao máximo não fazer com que ela se sinta ainda mais apreensiva em relação ao parceiro não querer vir, né? Eu sempre procuro dizer assim, quando eu pergunto por eles e elas respondem que está em casa, não quis vir porque estava dormindo e a consulta demorava: olha, não se preocupa com isso, é normal e sempre acontece do parceiro não vir.* (E9)

*Aqui tem muitos casos de sífilis, muito mesmo. Então, é importante essa captação pra tratar o parceiro nestes casos, mas é muito difícil também. Às vezes, acaba que não vem mais nem a mulher.* (E11)

### Indicadores de mudanças de paradigmas referentes à participação do parceiro no pré-natal

Os participantes relataram efeitos benéficos que observam em sua prática profissional, entre os quais estão o aumento da autoconfiança materna, a partilha de responsabilidades com o cuidado gestacional e, deste modo, a diminuição da sobrecarga sobre a mulher e o aumento do vínculo paterno com a criança e a família.

*A participação do parceiro dá até mais confiança pra* mãe, principalmente quando ela é mãe de primeira viagem, quando é muito jovem. E aqui tem bastante mãe muito jovem, que chegam bem nervosas, ansiosas*, a*í*, vindo com o parceiro,* dá uma segurança. A gente explica direitinho aos dois, dá as orientações direitinho. Aí eles também chamam a atenção delas, porque tem muitas mãezinhas que fogem da vacina, que não tomam medicamentos direitinho*, e eles estão lá supervisionando, auxiliando.* (E5)

*Eu tenho uma gestante que não entende nada do que eu explico, mas aí eu explicava pro parceiro, e aí ele, do jeito deles, explicava pra ela e ela entendia, geralmente tem muito isso também.* (E10)

*Eu tenho um exemplo aqui: um pai que, quando ele vem e eu coloco o sonar para ouvir o bebê, às vezes, eu não encontro. Aí o pai vai e conversa com o bebê, e daí eu consigo escutar o coraçãozinho do bebê. A mãe até falou que a bebê não pode ouvir a voz dele que, na hora que ela escuta, mexe.* (E6)

Os profissionais de enfermagem lançam mão de algumas estratégias para alcance das parcerias para realização do pré-natal para realizar a etapa de possibilitar a assistência ao público-alvo da estratégia. Entre elas, estão campanha de orientações sobre a importância da presença paterna durante o período gestacional para o desenvolvimento sadio da criança e adequação de horário, seja oferecendo consultas em horário noturno ou em horário de almoço. Outra estratégia foi a integração dos homens nas consultas, favorecendo sua participação em procedimentos que estabeleçam vínculo com a gestação e a criança, a exemplo da ausculta de batimentos cardíacos fetais (BCF).

*Eu acho que precisa ser feita uma grande campanha de esclarecimento para a comunidade, para a sociedade, da importância desse trabalho do homem junto* à *mulher que está grávida, né? Do pai do bebê.* (E4)

*Uma estratégia que utilizamos é a adequação do horário. Por exemplo, se ele está liberado às 11:30 para o almoço, eles até avisam e acabam sacrificando o horário do almoço pra poder estar aqui no horário da consulta. Só que isso é complicado pra eles, como acaba sendo complicado pra nós também, porque é difícil a gente trabalhar com uma agenda com horário, né? Mas dentro do possível, isso é uma estratégia que tem dado certo.* (E1)

*Eles gostam de estar presente quando a gente vai fazer ausculta do BCF, e a gente tem que aproveitar pra explicar pra eles que toda vez que ela vier pra consulta, nós vamos fazer a ausculta do BCF. E então aproveitamos para falar que o bebê escuta a voz do pai, que o bebê percebe as reações do pai. Então, o pai se sente participando desse processo.* (E4)

## DISCUSSÃO

O presente estudo identificou o absenteísmo dos homens durante o pré-natal, expressando as relações de gênero. Evidenciaram-se baixa sensibilização masculina para o cuidado, além de barreiras no acesso aos serviços de saúde, devido ao horário de funcionamento e rigidez das questões trabalhistas. A sobrecarga dos profissionais e as limitações estruturais contribuíram para maior ênfase na assistência materno-infantil. A presença do parceiro durante o pré-natal trouxe à tona manifestações de masculinidades hegemônicas durante os atendimentos, com a necessidade de o enfermeiro manejar as situações para promover a privacidade e o conforto da mulher e o envolvimento do homem no cuidado com os filhos. No entanto, evidenciaram-se benefícios do pré-natal do homem, na perspectiva dos enfermeiros, com aumento do vínculo e confiança da mulher, corresponsabilização no cuidado, desenvolvimento de atividades de educação em saúde com foco na atenção durante a gestação, parto e nascimento, puerpério e cuidados ao recém-nascido.

As representações culturais ancoradas no homem provedor, pouco presentes nos cuidados concepcionais por conta de seus horários ocupacionais, estão em convergência com os achados deste estudo, em que a baixa participação dos homens na assistência pré-natal também foi atribuída às limitações de tempo referentes ao trabalho^(18)^, desafios institucionais de empresas e das unidades de saúde, estruturas inadequadas, além de profissionais de saúde com preparação e formação inadequadas para lidar com esse público. Essa perspectiva reproduz a ideia de que o cuidado é uma função feminina, e representa barreira para a mudança de normas de gênero. Destaca-se, ainda, o papel pouco claro sobre como os homens acreditam ser importante estar envolvidos no período pré-natal, fatores socioculturais acerca dos papéis de gênero e a percepção de que as mensagens dos serviços de saúde são destinadas ao público de mulheres^(19)^.

Estudo realizado no Irã verificou que os homens têm pouco ou nenhum conhecimento sobre as mudanças físicas que ocorrem no ciclo gravídico-puerperal, nutrição adequada, atividades físicas indicadas, sexualidade, sinais de risco, alterações mentais e psicológicas na gravidez, parto, puerpério e cuidados neonatais^(20)^, o que evidencia a necessidade de participação desses, nas consultas com enfermeiros e profissionais de saúde com notável experiência em educação em saúde, para obter orientações que lhes atribuam autoconfiança no cuidado^(21)^ e possam ir de encontro às representações de masculinidade e paternidade hegemônicas. O perfil masculino atual é despreparado e inseguro sobre as expectativas que devem ser desempenhadas pelo pai^(22)^. Isto se deve, em partes, ao descontentamento e exclusão vindos de alguns agentes da prática que, ao prestarem os cuidados de assistência pré-natal, ignoram a presença dos parceiros durante as consultas, focando as orientações apenas à gestante, deixando o pai plano de fundo^(23)^.

Neste sentido, estudo de coorte prospectivo, realizado na Inglaterra, explorou, após o nascimento do primeiro filho, as opiniões e experiências de 42 pais em relação à saúde mental perinatal, e verificou que eles vivenciaram sofrimento psíquico, mas relutam em expressar suas necessidades de apoio^(24)^. Revisão integrativa também verificou que pais depressivos podem experimentar fortes sentimentos de inutilidade, culpa e menos confiança. Essa condição pode influenciar a resposta dos pais à gravidez, particularmente sua capacidade de vínculo com o feto. A depressão também inibiu o desenvolvimento de um vínculo positivo, pois os pais tendiam a ter pensamentos e emoções negativas em relação ao feto^(25)^. Esses resultados apontam a importância das necessidades de assistência à saúde mental de ambos os parceiros serem identificadas e gerenciadas pelos profissionais de saúde, com vistas ao bem estar psíquico da família.

Entre as barreiras descritas para o alcance dos parceiros no período pré-natal, estudo realizado em municípios da Amazônia brasileira verificou que a maioria das gestantes desconhece a estratégia pré-natal do parceiro e alega que não foram informadas pelos enfermeiros sobre a presença dos parceiros nas consultas^(22)^. Em contrapartida, em consonância com alguns dos achados desta pesquisa, a exemplo das mulheres optarem pela ausência do esposo à consulta, estudo realizado na Etiópia verificou que algumas mulheres mencionaram que a presença dos maridos nas unidades de saúde dificulta que elas expressassem seus sentimentos ou opiniões de forma livre e honesta. Assim, muitas vezes, as mulheres não comunicavam aos seus maridos quando os profissionais de saúde solicitavam a presença dos mesmos durante a consulta pré-natal subsequente. Isto condicionava a participação dos parceiros ao nível de relacionamento existente entre os casais e o conhecimento por parte dos profissionais das dinâmicas afetivas dos casais atendidos^(26)^.

O modelo biomédico existente raramente inclui informações direcionadas aos homens durante as consultas de pré-natal ou aos aspectos psíquicos e sociais. A falta de educação e conteúdo voltado para os homens pode deixar muitos pais com perguntas sem resposta e sentindo-se excluídos do processo^(22,23)^. Desse modo, as práticas profissionais demonstram persistência de práticas biologicistas e fragmentadas, forjadas a partir de percepções que reiteram a subalternidade de gênero, acompanhadas do não reconhecimento da responsabilidade da área para o enfrentamento de problemas sociais.

Destaca-se que os esquemas do inconsciente sexuado são resultados de estruturas históricas, altamente diferenciadas e nascidas de um espaço social, que se reproduzem através da aprendizagem ligada à experiência que os agentes - principalmente e, sobremaneira, os de prática - têm das estruturas do espaço. Isto quer dizer que o inconsciente se enraíza e se reproduz^(15)^. O enfrentamento do modelo do inconsciente sexuado, balizado na masculinidade hegemônica, demanda o seu reconhecimento pela saúde e áreas afins, e requer a superação de práticas fragmentadas, que desconsideram a integralidade entre biológico e social, bem como o protagonismo da enfermagem como agente transformador da realidade objetiva. A educação emancipatória desponta como possibilidade do desenvolvimento de estratégias de enfrentamento intersetorial, a partir da construção coletiva do conhecimento e de intervenções pautadas na equidade de gênero.

Os enfermeiros do sexo masculino frequentemente desafiam os estereótipos tradicionais de masculinidade, por meio de suas escolhas profissionais e comportamento no ambiente de trabalho^(27,28)^. Esses profissionais são treinados para serem cuidadosos, empáticos e atentos às necessidades das pessoas, famílias e comunidade, características muitas vezes associadas ao cuidado feminino na sociedade tradicional^(27)^. Assim, espera-se que esses profissionais apresentem uma visão mais flexível e progressista da masculinidade, que incorpore aspectos de cuidado e sensibilidade emocional, que podem ser exemplos positivos para a população.

Em contraste, a masculinidade entre os homens do território que acompanham ou não suas parceiras nas consultas de pré-natal pode variar amplamente, influenciada por fatores culturais, sociais e econômicos. A visão tradicional de masculinidade, em que o homem é visto como o provedor e a mulher como a principal cuidadora, ainda prevalece. Nessa visão, os cuidados de saúde, especialmente relacionados à maternidade, são frequentemente considerados responsabilidade feminina^(2)^. A participação dos homens no cuidado pré-natal tem o potencial para contribuir com a desconstrução e ruptura de normas tradicionais e patriarcais, demonstrando um compromisso com uma masculinidade mais inclusiva e participativa.

Neste estudo, foi possível observar o relato de absenteísmo nas consultas por parte dos parceiros. É possível verificar que, mesmo com a percepção da vontade do homem em participar do processo de gestação e de nascimento do filho^(29)^, no Brasil, ainda existem barreiras culturais e institucionais que o impedem de efetivar seu direito, a exemplo das diretrizes e legislações trabalhistas e preparo dos agentes da prática na assistência de porta de entrada dos serviços. O Artigo 473 da Lei nº 13.257/2016 referiu que o empregado poderia deixar de comparecer ao serviço sem prejuízo ao salário por até dois dias para acompanhar consultas médicas e exames complementares durante o período de gravidez de sua esposa ou companheira.

No entanto, destaca-se que este artigo de lei, editado no mesmo ano da publicação da Estratégia Pré-Natal do Parceiro^(8)^, tornou-se um impeditivo, portanto, paradoxal à participação do parceiro nas consultas pré-natais, uma vez que o próprio Ministério da Saúde do Brasil preconiza o mínimo de seis consultas pré-natais como modelo de assistência adequada. Somente em 2022, uma medida provisória (MP) ampliou o interstício para até seis dias de acompanhamento. Esta MP foi incluída e certamente pode ser compreendida em um contexto sociocultural de superação da masculinidade e paternidade hegemônica e, também, como incentivo a uma maior participação do homem na estrutura familiar e nas tarefas de cuidados^(30)^. Deve-se estreitar o vínculo entre a saúde e a construção de legislações trabalhistas que fomentem a parentalidade sadia, com possibilidade de intercâmbio entre as funções de cuidado^(2)^.

Elencam-se alguns elementos destacados na literatura como associados a maior participação dos parceiros nos cuidados pré-natais, como convite pela parceira, horários flexíveis, superestimar o risco de transmissão do HIV e outras infecções sexualmente transmissíveis (IST) de mãe para filho^(18)^, aceitando realizar testes rápidos para proteger parceiras e conceptos durante esse período vulnerável, integração do homem na consulta^(11,18)^. Estudo de método misto, realizado na Etiópia, demonstrou que trabalhar em cooperação com os líderes comunitários pode ser uma boa estratégia para influenciar o comportamento dos homens, e também são uma fonte de informação para os formuladores de políticas sobre estratégias para superar as barreiras aos resultados positivos em saúde materna, especialmente no nível da comunidade^(31,32)^.

Nos Estados Unidos, observou-se que o uso de aplicativos direcionados para introdução dos homens na educação pré-natal foi visto como positivo. Os participantes recomendaram a adição de módulos interativos, como uma ferramenta de planejamento financeiro e vídeos, para fortalecimento do aplicativo. Os homens se mostraram abertos às informações sobre como partilhar com suas parceiras os cuidados de uma gestação saudável e promover a saúde de seus filhos ainda não nascidos^(21)^. A visita domiciliar, com a integração dos parceiros, também pode ser utilizada como ferramenta estratégica. Os profissionais de saúde devem cuidar das mulheres grávidas na presença de seus companheiros. Também é recomendado que os casais sejam treinados juntos durante o período gestacional e o período pós-parto para os cuidados maternos e neonatais^(32)^. As experiências paternas são afetadas e há aumento de confiança, quando estes assumem e lidam com as responsabilidades do cuidado nos primeiros dias em suas casas^(6)^. Essas estratégias podem auxiliar nas fragilidades de captação de homens observadas nos resultados desta pesquisa.

Para além das estratégias dispostas, deve-se oportunizar que não somente ações de fomento ao vínculo entre adultos e crianças sejam criadas, nem somente o envolvimento do homem como instrumental ao cuidado da mulher, da criança e de oportunidade para que este realize exames preventivos de rotina, mas, principalmente, que possam ser construídas identidades articuladas à autorrealização das pessoas humanas^(2)^. A literatura infere que a construção da identidade paterna envolve um complexo trabalho subjetivo, consciente e inconsciente de elaborar as heranças recebidas dos próprios pais e transformá-las ao conceder o cuidado aos filhos, superando os modelos de representação de paternidade e masculinidade perpetrados ao longo de gerações e ressignificando a paternidade^(4,22)^.

A fim de romper um determinado paradigma, baseado em um parceiro que pouco participa da criação dos filhos e que negligencia o cuidado consigo, o Estado brasileiro deve prover meios de trazer uma nova representatividade da masculinidade e da paternidade, ao ancorar uma perspectiva de uma parentalidade ativa e de partilha de cuidados desde o período pré-concepcional, trazendo o homem para dentro das unidades de saúde, onde ele deve ser acolhido e envolto nos processos de cuidado, favorecendo a construção da paternidade e da formulação do vínculos familiares saudáveis.

### Limitações do estudo

Este estudo apresenta algumas limitações. A primeira delas é que foi realizado somente com a categoria profissional de enfermeiros, não proporcionando uma visão mais clara e coesa sobre a percepção dos agentes de práticas de saúde que compõem o Estado brasileiro. A segunda é que as respostas conduziram o estudo para uma perspectiva heteronormativa. Desse modo, discussões acerca dos aspectos pré-concepcionais, concepcionais e parentalidade entre casais homoafetivos foram dissipadas. No entanto, sobre este aspecto, a própria política de Estado estabelece o parceiro como alvo destinatário do cuidado, dissipando as distintas formas de parcerias envolvidas no processo de parentalidade. Além disso, é importante um olhar cauteloso para não tratar das representações aqui expressas de formas fixas, pois são fluídas às contínuas mudanças sociais. Salienta-se, também, que a enfermagem, enquanto categoria profissional, engloba um conjunto de indivíduos que diferem quanto aos modos de enxergar a vida, pois têm experiências individuais distintas e não podem ser compartimentados de forma engessada dentro de uma única esfera de suas vidas, neste caso, a categoria profissional a qual pertencem.

### Contribuições para as áreas da saúde, enfermagem ou políticas públicas

As campanhas de orientações sobre a importância da presença dos parceiros durante o período gestacional para o desenvolvimento saudável da criança devem ser fomentadas e financiadas pelas políticas públicas de Estado. Estudos sobre viabilidade de ampliação do período de atendimento das UBS em horário não comercial devem ser realizados.

Os componentes curriculares voltados à formação de enfermeiros devem abordar as questões nocivas da masculinidade hegemônica para a sociedade como um todo. Primordialmente, deve ser promovida a construção de identidades parentais de cuidado articulada à autorrealização das pessoas humanas.

Além disso, a implementação de programas de apoio específicos para homens, como grupos de pais ou sessões de aconselhamento, pode ajudar a abordar as preocupações e inseguranças que muitos homens podem ter sobre seu papel durante a gravidez. Esses programas podem fornecer informações práticas e emocionais, ajudando a normalizar a participação masculina no cuidado pré-natal.

Incorporar essas reflexões sobre masculinidade nas práticas de enfermagem não só melhora a qualidade do cuidado pré-natal, mas também contribui para a construção de uma sociedade mais equitativa e inclusiva. Envolvendo ativamente os homens no cuidado, os enfermeiros podem promover uma cultura de saúde onde todos os membros da família são valorizados e apoiados em suas necessidades e contribuir para o alcance dos ODS.

## CONSIDERAÇÕES FINAIS

Apesar do reconhecimento dos profissionais da importância do pré-natal do homem, persistem as questões de gênero no cotidiano do atendimento na comunidade. A assistência pré-natal foi representada fortemente por marcadores de masculinidade hegemônica, caracterizada por absenteísmo do parceiro, motivada por questões macros e micro referentes à construção da paternidade, a exemplo da dificuldade de justificar ausência de homens para participação de consultas pré-natais em serviços laborais e, no âmbito doméstico, o parceiro optar por não comparecer ou, ainda, comparecer, mas terceirizar o cuidado aos filhos à figura de mulheres mesmo durante a consulta, a exemplo da avó. Os papéis de gênero foram reforçados pelos profissionais da prática, e o cuidado foi naturalizado como inerente aos papéis femininos. Os aspectos negativos referentes à participação do parceiro também estiveram imbricados com representações de masculinidade hegemônica, a exemplo da pouca privacidade das mulheres para se queixar dos parceiros aos profissionais de saúde. A sobrecarga de trabalho de enfermeiros na APS foi imbricada com a priorização de aspectos fisiopatológicos maternos e infantis em detrimento dos benefícios biopsicossociais do acolhimento e fomento da integralidade do parceiro no cuidado. Ainda, os horários de atendimento das equipes, bem como as severas condições trabalhistas, permanecem como desafios. Esses elementos relacionam-se às dimensões conceituais da formação profissional, direcionadas para atender à produção de demandas com foco nas perspectivas biológicas e também representadas em modelos de masculinidade e feminilidades hegemônicas. Assim, há lacunas nas estratégias de captação e adesão da saúde do homem e, consequentemente, da construção de uma masculinidade, paternidade e parentalidade acolhedoras e com força motriz para desempenhar transformações estruturadas.
